# Spatial variability in levels of benzene, formaldehyde, and total benzene, toluene, ethylbenzene and xylenes in New York City: a land-use regression study

**DOI:** 10.1186/1476-069X-11-51

**Published:** 2012-07-31

**Authors:** Iyad Kheirbek, Sarah Johnson, Zev Ross, Grant Pezeshki, Kazuhiko Ito, Holger Eisl, Thomas Matte

**Affiliations:** 1 New York City Department of Health and Mental Hygiene, Bureau of Environmental Surveillance and Policy, 125 Worth Street, CN34E, New York, NY 10013, USA; 2 ZevRoss Spatial Analysis, 120 N. Aurora St, Suite 3A, Ithaca, NY 14850, USA; 3 Center for the Biology of Natural Systems, Queens College, 65-30 Kissena Blvd, Remsen Hall 311, Flushing, NY 11367, USA

**Keywords:** Benzene, Formaldehyde, BTEX, Land use regression (LUR), Air toxics, Traffic, Hazardous air pollutants (HAP)

## Abstract

**Background:**

Hazardous air pollutant exposures are common in urban areas contributing to increased risk of cancer and other adverse health outcomes. While recent analyses indicate that New York City residents experience significantly higher cancer risks attributable to hazardous air pollutant exposures than the United States as a whole, limited data exist to assess intra-urban variability in air toxics exposures.

**Methods:**

To assess intra-urban spatial variability in exposures to common hazardous air pollutants, street-level air sampling for volatile organic compounds and aldehydes was conducted at 70 sites throughout New York City during the spring of 2011. Land-use regression models were developed using a subset of 59 sites and validated against the remaining 11 sites to describe the relationship between concentrations of benzene, total BTEX (benzene, toluene, ethylbenzene, xylenes) and formaldehyde to indicators of local sources, adjusting for temporal variation.

**Results:**

Total BTEX levels exhibited the most spatial variability, followed by benzene and formaldehyde (coefficient of variation of temporally adjusted measurements of 0.57, 0.35, 0.22, respectively). Total roadway length within 100 m, traffic signal density within 400 m of monitoring sites, and an indicator of temporal variation explained 65% of the total variability in benzene while 70% of the total variability in BTEX was accounted for by traffic signal density within 450 m, density of permitted solvent-use industries within 500 m, and an indicator of temporal variation. Measures of temporal variation, traffic signal density within 400 m, road length within 100 m, and interior building area within 100 m (indicator of heating fuel combustion) predicted 83% of the total variability of formaldehyde. The models built with the modeling subset were found to predict concentrations well, predicting 62% to 68% of monitored values at validation sites.

**Conclusions:**

Traffic and point source emissions cause substantial variation in street-level exposures to common toxic volatile organic compounds in New York City. Land-use regression models were successfully developed for benzene, formaldehyde, and total BTEX using spatial indicators of on-road vehicle emissions and emissions from stationary sources. These estimates will improve the understanding of health effects of individual pollutants in complex urban pollutant mixtures and inform local air quality improvement efforts that reduce disparities in exposure.

## Background

Despite regulatory controls, urban populations are exposed to toxic air pollutants with potential to cause cancer or other serious health effects. The 1999 Amendments to the Clean Air Act identified 187 hazardous air pollutants (HAPs) subject to emissions based controls due to health effects associated with ambient exposures [1]. These regulations include controls on 174 stationary source categories to meet maximum achievable control technology standards and mobile source air toxics rules that reduce vehicle emissions through fuel controls, including lowering limits on benzene in gasoline beginning in 2011 [2].

HAPs commonly found in urban areas include formaldehyde and a group of aromatic volatile organic compounds (VOC): benzene, toluene, ethylbenzene, xylene (together known as BTEX). Among these, benzene and formaldehyde are classified by the International Agency for Research on Cancer as human carcinogens (Group 1); both are key drivers of estimated cancer risk from organic HAPs in the US [3,4]. Other BTEX compounds--toluene, ethylbenzene, and xylene--have been found to produce adverse health effects including respiratory and neurological effects [5-7] and react to form secondary organic aerosols, contributing to ambient fine particulate matter (PM_2.5_) [8]. BTEX and formaldehyde also play important roles in the photochemical reactions that form ozone [9].

Recent analyses suggest that 49% of New York City residents live in census tracts exceeding the 1 in 10,000 HAP-attributable cancer risk benchmark compared to 4.8% of the population nationwide, with the majority of the risk attributed to benzene and formaldehyde exposures [10,11]. Primary local sources of BTEX are on-road and non-road gasoline vehicles and engines, with emissions from petroleum transport/storage and solvent usage also making substantial contributions [12]. On- and non-road gasoline and diesel vehicles and engines are also predominant sources of primary formaldehyde emissions in NYC with additional contributions from stationary-source fuel combustion [12]. Formaldehyde is also formed secondarily by photooxidation of hydrocarbons. Ambient formaldehyde levels in New York City have been observed to peak in summer months, likely due to seasonal increases in photochemical activity [13].

While national air toxics regulations have reduced exposures, the limited number of monitoring sites in urban areas restricts the ability to assess spatial variation in concentrations within cities for developing local control policies. For example, in New York City there are currently six regulatory monitors reporting VOC measurements and five reporting aldehydes, with monitors operating only every sixth day [14]. While this network provides valuable information on air toxic trends useful in evaluating exposure and regulating ozone, they are not sufficient to understand fine scale intra-urban spatial variation in concentrations due to localized sources such as traffic [15,16].

Recently, land-use regression (LUR) models have been increasingly used to estimate intra-urban spatial variability of air pollutants and in developing exposure estimates for epidemiological research [17,18]. They have been used in New York City to develop exposure estimates for fine particulate matter (PM_2.5_), oxides of nitrogen (NO_x_), and sulfur dioxide (SO_2_) (Clougherty et al. submitted 2011, [19]). While many LUR studies focus on nitrogen dioxide NO_2_ and PM_2.5_, they have also been used to estimate BTEX concentrations [16,20-23].

This paper evaluates spatial variation in benzene, total BTEX and formaldehyde concentrations across New York City using a saturation sampling campaign conducted in the spring of 2011 and land-use regression modeling.

## Methods

### Spatial and temporal allocation of sites

BTEX and formaldehyde monitoring was conducted at a subset of the 150 sites routinely monitored for PM_2.5_, elemental carbon, PM_2.5_ constituents, NO_x_, SO_2_ and ozone throughout NYC as part of the New York City Community Air Survey (NYCCAS) network, an initiative within the City’s sustainability plan, PlaNYC [24]. The NYCCAS monitoring network sites were selected to capture the range in variation of key local emissions sources while providing adequate spatial coverage throughout the City. A description of the selection process for these 150 sites is described elsewhere (Matte et al. submitted 2011). In short, 120 sites were selected for monitoring through stratified random sampling of 7,756 300 m x 300 m grid cells with oversampling in areas of high traffic and high building density- indicators of two categories of important local emissions sources- to account for skewed distributions of these source proxies within New York City. We chose building density rather than population density as an indicator of source activity suitable for both residential and commercial areas of the city. Thirty additional sites were selected to fill spatial gaps and capture areas of interest.

**Figure 1:**
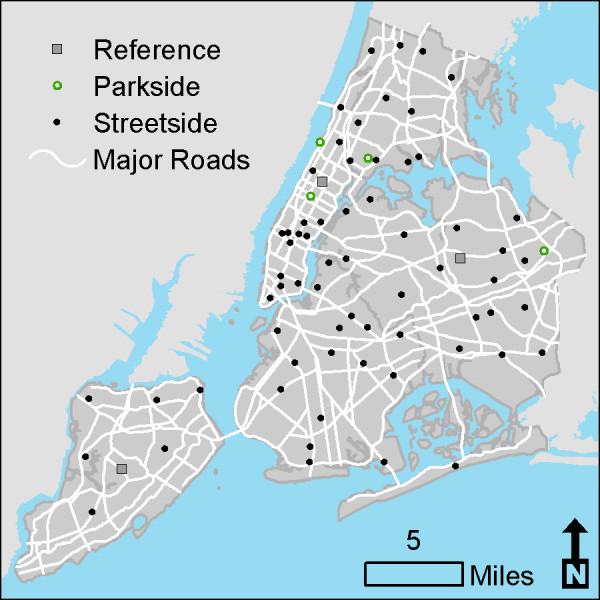
Map of New York City Community Air Survey sites monitored for BTEX compounds and formaldehyde.

Of the original 150 sites, we selected 70 sites for air toxics monitoring (referred to as “distributed” sites) by first retaining 21 sites that were geographically isolated from other monitoring locations or had produced high residuals in our prior statistical models for NO_x_, SO_2_, PM_2.5_, and EC. These sites were included to ensure that the monitoring captured a full range of traffic and land-use settings. We then randomly selected from the remaining available sites. We compared the distributions of these 70 sites in relation to traffic and building density to the distribution in the original 150 sites to confirm that similar coverage of major source density was achieved in the subset of sites selected for air toxics monitoring (Table 1). Three reference sites were selected in parks, away from major sources, in Central Park in Manhattan, Queens College in Queens, and La Tourette Golf Course in Staten Island (Figure 1).

**Table 1:** Distribution of traffic and building density at NYCCAS network sites and Air Toxics sampling sites

		**Air Toxics**		**Full NYCCAS**	
		**(n = 70)**		**(n = 150)**	
**Building Density**	**Traffic Density**	**Count**	**Percent**	**Count**	**Percent**
High	High	16	23%	34	23%
Norm	High	14	20%	35	23%
High	Norm	20	29%	36	24%
Norm	Norm	20	29%	45	30%

High is defined as highest quartile of citywide 300 m X 300 m lattice values.

We collected samples of BTEX and formaldehyde at each of the 70 distributed sites, 14 of which were allocated at random to each of five two-week sessions, from 3/22/2011 to 6/1/2011. At the three reference sites, samples were collected during all five sessions to assess city-wide temporal variation related to meteorology.

### Air sampling and analysis

Formaldehyde and BTEX compounds were measured with Radiello radial passive sampling tubes (Fondazione Salvatore Maugeri, Padova, Italy). Samplers were placed in weather protective shelters and mounted at 10 feet onto street-side signal and lamp posts. Formaldehyde measurements were taken for 1-week while BTEX measurements were conducted for 2-weeks to meet sampler manufacturer’s sample time specifications [25,26].

Passive BTEX samplers contained activated charcoal that collects VOCs by adsorption. Sample analysis was conducted by Air Toxics Limited (Folsom, CA) by extraction with carbon disulfide and analyzed using gas chromatography with mass spectrometry (GCMS). GCMS identified five BTEX compounds: benzene, toluene, ethylbenzene, *o*-xylene, and *m/p*-xylene, which were summed to compute the total BTEX concentration. These samplers have been used in VOC field monitoring campaigns [27-29] as well as prior LUR studies [20].

Passive aldehyde samplers contained 2,4-dinitrophenylhydrazine (2,4-DNPH) coated silica which converts aldehydes to stable hydrazone derivatives, 2,4-dinitrophenylhydrazone. Sample analysis was performed by Air Toxics Limited (Folsom, CA) by extracting hydrazones with acetonitrile and analyzing using reverse phase high-pressure liquid chromatography with ultra-violet detection at 360 nm (HPLC-UV). Passive sampling by 2,4-DNPH derivitaziation has been evaluated and applied extensively in ambient formaldehyde monitoring studies [30-32].

### Quality assurance

During each sampling session one field blank was placed unopened at the La Tourette reference site for the duration of the session and analyzed alongside all other samplers. At two sites in each session, two sets of samplers were deployed side by side to assess differences in collocated samplers. Laboratory quality control procedures followed guidelines established for passive VOC and aldehyde monitoring by the sampler manufacturer using standard EPA and OSHA methodologies [33,34]. For each pollutant, descriptive statistics were computed by session to identify potential outliers for further investigation.

### Data analysis

#### Descriptive analysis

We computed descriptive statistics across all distributed and reference site measurements and compared concentrations to those reported during the same time period at rooftop regulatory monitors [14]. Raw measurements were then adjusted for temporal variation by dividing the distributed site measurements by the mean reference value in each session then multiplying this ratio by the mean of reference sites across the entire period. We described spatial variability by computing the coefficient of variation (CV) of temporally adjusted measurements across all sessions. We examined spatial distributions within each session by computing the CV (based on unadjusted values) within each session and examining plots of monitored concentrations, session means, and reference site means. To assess temporal variation, we regressed raw distributed site concentrations on session-specific means of reference sites, and used the R-squared (R^2^) as the indicator of temporal variation (referred to as “temporal R^2^” in Results section).

#### Geographic variables

Spatial data on emission source indicators were collected and analyzed using ArcGIS 9.2 (ESRI, Redlands CA). These datasets were obtained from a variety of public and private sources and encompassed a range of data types and resolution from highly resolved road network line data to traffic volume modeled along “links” between destinations. Source indicator categories included total and road-specific measures of traffic, mobile source diesel combustion, population metrics, built space area, land-use type, and emissions permits from point sources, transportation facilities, and waste treatment and transfer facilities (Table 2). City-issued permits on point sources were filtered by searching the business description field using keywords derived from the EPA National Emissions Inventory [12] of processes known to produce the air toxics of interest. For each indicator, covariates were calculated within 15 buffers surrounding each monitoring location, at distances of 50 to 1000 meters. Detailed descriptions of the GIS datasets used to develop source indicators for NYCCAS analyses are available in Additional file 1: Table S1.

**Table 2:** Summary of GIS-based source indicators

**Source Category**	**Variables**	**Data Sources**
Traffic Indicators	Un-weighted and kernel-weighted road and traffic density, number of signaled intersections, distance to and characteristics of nearest roadway	New York Metropolitan Transportation Council, Highway Performance Monitoring System, Accident Location Information System, Market Planning Solutions TrafficMetrix data, NYC Department of Transportation Truck Routes
Population Metrics	Census population density, LandScan population density	2000 US Census, Oak Ridge National Laboratory LandScan^TM^
Built Space	Density of built space by land use category	NYC Department of City Planning Primary LandUse Tax Lot Output (PLUTO™)
Permitted Emissions	Permitted combustion sources, solvent use industries (excluding dry cleaning), petroleum bulk storage locations	NYS Department of Environmental Conservation, NYC Department of Environmental Protection
Transportation and waste transfer facilities	School bus depots, waste transfer stations, wastewater treatment facilities, marine terminals, airports	NYC Department of Citywide Administrative Services, NYC Department of Education, NYC Department of Sanitation, NYC Office of Emergency Management

Calculated within 50 m buffers between 50 to 500 meters and 100 m buffers between 500 to 1000 meters.

#### LUR model building process

Prior to modeling, concentrations among the three reference sites across the five sampling sessions were examined for similarity in temporal patterns. For benzene, while two reference sites were highly correlated (Pearson’s Correlation (r) = 0.84), one site showed low correlation with the others (r = 0.13 and −0.18) potentially indicating local source influence on temporal variation at that specific site. This site’s benzene measurements were removed to avoid distortion or bias in temporal adjustment. Raw concentrations were then used as the dependent variable in the model building process and each session’s mean pollutant concentration at the reference sites was added as a covariate [35] to adjust for city-wide temporal variation due to meteorology while explicitly accounting for error in estimating the temporal term.

Source indicator variables were grouped into six emission indicator-based categories: total traffic density, truck and bus traffic, permitted combustion-related emissions from point sources, built space density, population density, non-combustion permitted emissions (solvent use, petroleum/chemical bulk storage). For each pollutant, we used a Pearson’s correlation matrix to select the two buffer specific variables within each category most correlated with temporally adjusted pollutant concentrations. Each of these two variables was paired with a second category-specific term that optimized the R^2^ in a two-variable model against the pollutant concentration. This resulted in a total of four candidate covariates per category that were considered in subsequent model building.

We followed a manual forward step-wise model-building process using reference site concentrations, emissions source covariates, and site characteristics. Models were first fit using a randomly selected “modeling subset” of 85% (n = 59) of distributed sites and the resulting provisional models were validated by comparing predicted values with measured values at the remaining 15% (n = 11) of sites. Model diagnostics, including studentized residuals and Cook’s distance values, were inspected for outliers and highly influential points and models were evaluated for coherence with known emission source patterns and for sensitivity to alternative emission source indicators. Once the provisional models were validated, raw measurements from all 70 sites were used to produce final model parameters describing the spatial and temporal variability in pollutant concentrations and for predictions of seasonal mean values. After building the final model we computed an additional purely spatial model that regressed the temporally-adjusted pollutant concentrations onto the final set of spatial source terms to confirm that both temporal adjustment strategies produced comparable results. The overall fit of this model is reported in the Results section as the amount of spatial variability explained by the model.

## Results

### Descriptive statistics

Across 10 weeks of monitoring, 70 sites were sampled successfully for formaldehyde while 69 of 70 scheduled sites were sampled successfully for BTEX compounds due to a field error where a sampler was not deployed to one site scheduled for monitoring. Measurements in all samples exceeded the limit of quantification (LOQ) for BTEX compounds and formaldehyde. Field blank concentrations were below the LOQ for all BTEX compounds and all but one formaldehyde sample. Collocated samples (n = 10) showed good agreement with mean absolute percent differences of 10.9%, 8.0%, and 4.6% and R^2^ of 0.80, 0.94, and 0.98 for benzene, BTEX, and formaldehyde, respectively. One formaldehyde result was removed from the analysis because of implausibly high concentrations. This yielded 69 total benzene, BTEX and formaldehyde samples from distributed sites used in further analyses.

Street-side concentrations of all pollutants were higher on average than reference site concentrations while average benzene and BTEX levels at distributed sites showed higher concentrations and wider ranges than those reported at regulatory monitoring sites during the same period (Table 3). Average formaldehyde levels from distributed sites were slightly lower than average regulatory site measurements due to one regulatory monitor reporting high concentrations for several days during the campaign.

**Table 3:** Summary statistics for pollutant concentrations at NYCCAS sites and rooftop regulatory monitoring sites from 3/22/2011-6/1/2011

	**Distributed Sites**			**Reference Sites**			**Regulatory Sites**		
	**n**	**Mean (μg/m**^3^**)**	**Range (μg/m**^3^**)**	**n**	**Mean (μg/m**^3^**)**	**Range (μg/m**^3^**)**	**n**	**Mean (μg/m**^3^**)**	**Range (μg/m**^3^**)**
Benzene	69	0.82	0.34-2.3	3	0.52	0.50-0.58	6	0.65	0.50-0.76
BTEX	69	4.66	1.52-20.4	3	2.35	2.05-2.72	6	3.58	2.58-4.97
Formaldehyde	69	2.21	1.20-3.70	3	1.83	1.62-2.04	5	2.33	1.16-4.31

**Figure 2:**
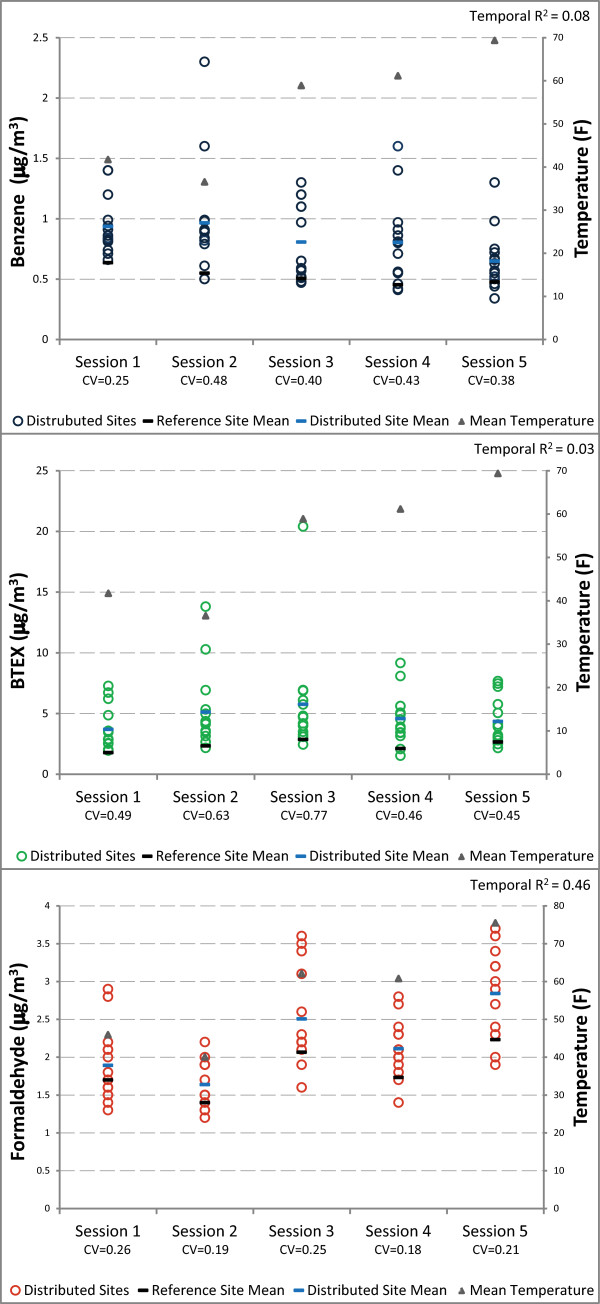
Distribution of two-week average benzene and BTEX and one-week average formaldehyde concentrations with average session temperatures measured at monitoring sites.

Spatial variability, estimated by the CV across all temporally adjusted measurements, was greatest for BTEX, followed by benzene, then formaldehyde (CV of 0.57, 0.35, 0.22, respectively). Benzene and BTEX concentrations showed little temporal variation; 8% and 3% of variance, respectively, was explained by session (Figure 2). Formaldehyde showed the most city-wide temporal variability (temporal R^2^ = 46%), with levels generally increasing as the season progressed and temperatures increased (Figure 2). Temporally adjusted concentrations were spatially correlated across all three pollutants with slightly better correlation between benzene and total BTEX or formaldehyde (r = 0.73) than formaldehyde and BTEX (r =0.69).

### Modeling results

#### Benzene

**Figure 3:**
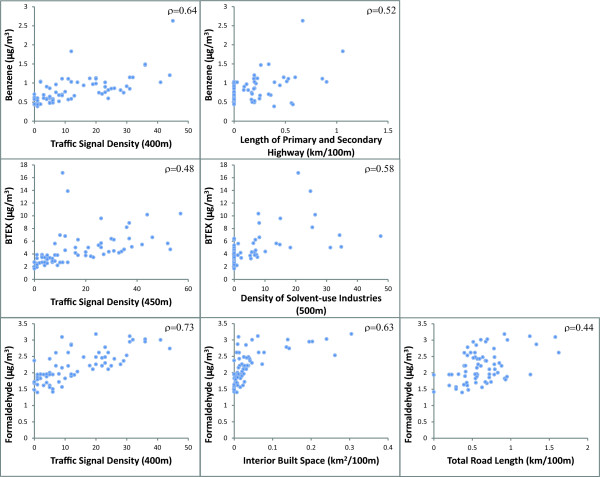
Scatterplots of GIS covariates and temporally adjusted concentrations.

**Figure 4:**
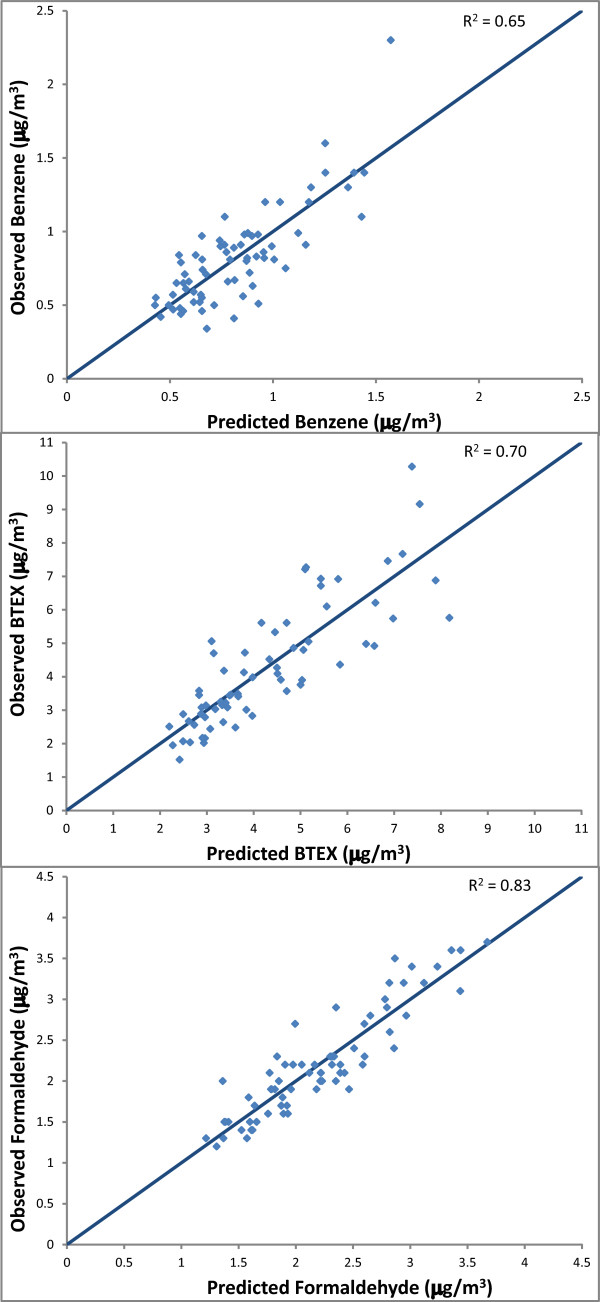
Comparisons of temporally adjusted observed measurements vs. LUR predicted estimates at monitoring sites.

Predicted concentrations from the provisional model explained 62% of the variance in concentrations at the validation sites. Spatial and temporal variability of benzene was associated with, in order of importance based on partial R^2^, traffic signal density within 400 m of the monitors, length of interstate, state, and county highways within 100 m, and the reference site mean. The bivariate relationships between the spatial model terms and temporally adjusted concentrations demonstrated consistent positive associations across all 69 monitoring sites (Figure 3). Including all 69 sites in the final model showed that after controlling for other model terms, an inter-quartile range (IQR) increase in traffic signal density (an indicator of vehicle traffic and congestion) was associated with an increase in benzene concentration of 0.32 μg/m^3^ while an IQR increase in road length was associated with an average increase in benzene of 0.15 μg/m^3^. These terms describe 60% of the spatial variability (not shown) of benzene across all monitoring sites and, together with the reference site means, 65% of the temporal and spatial variation in benzene (Table 4, Figure 4).

**Table 4:** **Land-use regression model results for benzene, BTEX, and formaldehyde. Final model terms listed in order of importance based on partial R**^**2**^

				**R**^2^	
**Covariate**	**Coefficient**	**Standard Error (SE)**	**p-value**	**Model**	**Partial**
**Benzene (n = 69)**				0.65	
Intercept	0.052	0.188	0.783		--
Number of signals within 400 meters	0.017	0.002	<.0001		0.36
Length of interstate, state, and county highways within 100 meters (km)	0.591	0.101	<.0001		0.18
Reference site mean	0.799	0.340	0.022		0.03
**BTEX (n = 67)**				0.70	
Intercept	0.568	0.801	0.481		--
Number of signals within 450 meters	0.074	0.009	<.0001		0.34
Kernel-weighted smooth of solvent-based industry locations (500 meter radius)	0.072	0.013	<.0001		0.14
Reference site mean	0.873	0.328	0.010		0.03
**Formaldehyde (n = 69)**				0.83	
Intercept	−0.725	0.224	0.002		--
Reference site mean	1.209	0.119	<.0001		0.28
Number of signals within 400 meters	0.020	0.004	<.0001		0.07
Road length within 100 meters (km)	0.561	0.112	<.0001		0.07
Built space within 100 meters (km^2^)	2.477	0.716	0.001		0.03

#### BTEX

Two sites showed high studentized residuals (>8) and high Cook’s distance values (>0.6) potentially indicating unusual emissions patterns near the site. These sites, located in the industrial areas of the South Bronx, were not outliers for benzene and formaldehyde, but showed very high levels of toluene, ethylbenzene, and the xylenes. To avoid distortion of the final, city-wide model, we elected to remove these sites from the final model. Predicted concentrations from the provisional model explained 65% of the variance in concentrations at the validation sites. The bivariate relationships between these spatial model terms and temporally adjusted concentrations confirmed that consistent positive associations were observed across all 67 sites (Figure 3). Spatial and temporal variability of BTEX compounds was associated with, in order of importance based on partial R^2^, traffic signal density within 450 m of the monitors, kernel-weighted density of solvent-use industries within 500 m, and reference site mean. The final model that included all 67 sites showed an IQR increase in traffic signal density was associated with an increase in BTEX concentration of 1.62 μg/m^3^ while an IQR increase in density of permitted solvent-use industries was associated with an increase in BTEX concentration of 0.52 μg/m^3^. These terms described 64% of the spatial variability (not shown) in BTEX across all monitoring sites and, in combination with the reference site means, explained 70% of the spatial and temporal variation in BTEX (Table 4, Figure 4).

#### Formaldehyde

Predicted concentrations from the provisional model explained 68% of the variance in concentrations at the validation sites. Spatial and temporal variability of formaldehyde was associated with, in order of importance based on partial R^2^, reference site mean, traffic signal density within 400 m of the monitors, length of roads within 100 m, and interior built space within 100 m. The bivariate relationships between these spatial model terms and temporally adjusted concentrations demonstrated consistent positive associations across all 69 monitoring sites (Figure 3). The final model that included all 69 sites showed an IQR increase in signal density was associated, on average, with an increase of 0.36 μg/m^3^ formaldehyde, an IQR increase in interior built space density (index of amount of fuel combustion for heating) was associated with an increase of 0.08 μg/m^3^, and an IQR increase in road density was associated an increase of 0.19 μg/m^3^. These terms described 69% of the spatial variation (not shown) in formaldehyde across all monitoring sites, and in combination with the reference site means, they described 83% of the spatial and temporal variation (Table 4, Figure 4).

## Discussion

This study demonstrates significant intra-urban spatial variability in ambient levels of benzene, total BTEX, and formaldehyde across New York City monitoring sites, with the widest range in concentrations found in total BTEX. Within the season, we observed limited temporal variability for benzene and BTEX while formaldehyde levels increased with increasing average temperatures. Land-use regression models explained 65%, 70%, and 83% of the total variability of benzene, BTEX, and formaldehyde, respectively with temporal terms and spatial variables representing traffic density, solvent-use industries and built space. The provisional models built with the modeling subset were found to predict concentrations well, predicting 62% to 68% of monitored values at validation sites.

Average benzene and BTEX levels were higher than those measured at rooftop regulatory monitors during the study period, reflecting closer proximity of NYCCAS monitoring sites to traffic sources. Prior NYC-based monitoring studies of air toxics showed higher ambient levels of benzene and BTEX at residential sites mainly in the Bronx and Northern Manhattan than levels reported here [13,36]. This is likely explained by overall decreases in concentrations in NYC and nationwide over the past decade as well as relatively higher levels of traffic related pollutants in Northern Manhattan and the Bronx compared to the city overall [14,37]. Associations of benzene and BTEX concentrations with high traffic density are consistent with prior monitoring studies [23,38,39].

We found that variables specific to traffic congestion and volume best explained the spatial variability of benzene, with traffic volume indicated through total road lengths around monitoring sites and indicators of traffic density and congestion represented by traffic signal density. These variables were consistent with known sources of benzene in NYC, where gasoline vehicles are, collectively, the predominant source [12]. Prior LUR models for benzene have shown similar results, although some included additional terms related to petroleum usage, proximity to point sources, and population density [16,21-23]. The association of benzene concentrations with traffic within 400 meters of monitoring locations is consistent with observations that increased benzene levels near roadways decay to background within around 300 meters [40]. In contrast to many prior LUR studies, we chose to address temporal variation by using raw unadjusted concentrations as the dependent variable and the reference site mean as a covariate with the spatial covariates in the model. The advantage of this approach over a model in which temporally adjusted values are regressed onto spatial covariates is that, in estimating the slope for emission source terms, it adjusts for city-wide temporal variation due to meteorology while explicitly accounting for error in estimating the temporal term.

The correlates of spatial variability in total BTEX we observed in New York City are also consistent with known local emission sources including traffic and solvent usage [12] and with prior studies linking higher BTEX concentrations to traffic as well as distance to VOC emitting point sources [20,21,41]. Likely due to limited geographic distribution throughout the city, we did not find associations with large point sources reported in the National Emissions Inventory [12] and Toxics Release Inventory [42] or petroleum storage facilities. We did however find associations with density of nearby facilities too small to require Title V permits, but permitted by the City to use solvents in industries known to produce BTEX compounds such as spray booths, graphics industries, and auto body and detailing shops. These facilities are distributed throughout many neighborhoods, although more concentrated in industrial areas. An important limitation of our data is the lack of detailed information on solvent type and quantity at these smaller permitted facilities. Additional sampling near different types of facilities and improved emissions data or proxies could help elucidate these patterns in future work.

Formaldehyde measurements showed less spatial variability than benzene and total BTEX, compatible with findings from prior intra-urban analyses of data from national monitoring networks [43]. We found more temporal variability in formaldehyde with levels increasing with higher average temperatures. These findings are consistent with studies indicating higher temperature and longer daylight hours increase photochemical formation of secondary formaldehyde and levels peak during warm months and mid-day periods [43-45]. To our knowledge there have been no published LUR models for formaldehyde. The predictors of spatial variation found are consistent with known sources of local primary ambient formaldehyde with higher levels found in areas of increased traffic emissions and interior built space indicating increased fuel combustion related to space and water heating.

This study indicates that LUR modeling can be applied successfully to predicting benzene, BTEX, and formaldehyde levels for use in exposure assessment and epidemiological research in complex urban environments like New York City. Prior VOC and aldehyde exposure assessments have applied modeled data from EPA’s National Air Toxics Assessment (NATA) [3,46-48], regulatory monitoring data [49,50], and combinations of fixed site and personal monitoring [13,41]. While NATA modeling is useful in estimating relative concentrations in regional scale assessments, in fine scale, urban analyses, estimates are subject to limited spatial resolution of area and mobile sources in the National Emissions Inventory [51]. Similarly, using few central-site regulatory monitors for exposure classification limits the ability to assess near source concentration gradients, such as near roadways [15]. Prior air toxics assessments conducted in New York City using fixed site and personal monitoring have provided important data on indoor, outdoor, and personal exposures among cohorts in specific neighborhoods [13,36] but have not offered comprehensive assessments across the City.

City-wide average temporally adjusted springtime measurements of benzene correspond to concentrations between EPA’s 1 in 10^5^ and 10^6^ lifetime cancer risk benchmarks [52]. Average formaldehyde levels in this study correspond to concentrations above the EPA 1 in 10^5^ lifetime cancer risk benchmark [53]. While risk benchmarks are based on continuous exposures experienced over a lifetime, these springtime results suggest HAPs may contribute meaningfully to cancer and other health risks among large populations of New Yorkers who reside in close proximity to traffic and other local emission sources.

An important limitation to these results is that data was collected during a single spring season. Pollutant concentrations observed may differ in other seasons, particularly for formaldehyde where differences in photochemical activity will affect secondary formation. However, spatial variation should be consistent throughout the year as patterns in source density overall remain relatively unchanged over short time periods. As with all LUR studies, limited data on specific emitters of VOC compounds adds uncertainty to model estimates and likely attenuates associations between observed concentrations and source indicators.

These findings, and those from prior saturation sampling and land-use regression studies conducted in New York City (Clougherty et al. submitted 2012, [19,37]), indicate many of the neighborhoods impacted by high levels of PM_2.5_ and NO_2_ exposure may also experience high levels of benzene, BTEX and formaldehyde. High traffic density contributes to higher levels of both criteria and toxic pollutants evaluated here while areas of high building density are associated with high PM_2.5_ and formaldehyde levels. Because most studies of intra-urban spatial variation in air pollution exposures have focused on criteria pollutants, characterizing spatial patterns of exposure to common urban air toxics will be valuable in elucidating the health effects of individual pollutants in common pollutant mixtures [54] as well as development of emissions reduction strategies that maximize health benefits.

## Conclusions

In this analysis we used high density air quality monitoring and land-use regression methods to estimate variability in ambient exposures to benzene, BTEX compounds, and formaldehyde in New York City. We found significant intra-urban spatial variability in all compounds. Indicators of motor vehicle traffic, solvent usage, and stationary source combustion explained much of the variability in concentrations of these air toxics. Many of the same neighborhoods identified by prior studies as being impacted by high levels of criteria air pollutants are also found to have relatively higher levels of these common air toxics due to shared local sources. Characterization of these spatial patterns in air toxics will help improve understanding of the health effects of individual pollutants in complex urban air pollution mixtures and develop targeted air quality management strategies that reduce health disparities in pollutant-attributable adverse health outcomes.

## Abbreviations

BTEX, Sum of Benzene, Toluene, Ethylbenzene, Xylenes; CV, Coefficient of Variation; 2,4-DNPH, 2,4-dinitrophenylhydrazine; EPA, U.S. Environmental Protection Agency; GCMS, Gas Chromatograph with Mass Spectrometry; HAP, Hazardous Air Pollutant; HPLC-UV, High Pressure Liquid Chromatography with Ultra-Violet detection; LUR, Land-Use Regression; NATA, National Air Toxics Assessment; NO2, Nitrogen Dioxide; NOx, Oxides of nitrogen; NYCCAS, New York City Community Air Survey; OSHA, Occupational Health and Safety Administration; PM2.5, Particulate Matter with aerodynamic diameter less than or equal to 2.5 micrometer; r, Pearson’s correlation coefficient; R2, R-squared; SO2, Sulfur Dioxide; VOC, Volatile Organic Compounds; WHO, World Health Organization.

## Competing interests

The authors declare they have no competing financial interests.

## Authors’ contributions

IK contributed to study design, data collection and analysis, and drafted and edited the manuscript. SJ and ZR conducted the statistical analysis and contributed to manuscript drafting and editing. SJ, ZR, and GP contributed to developing the GIS data layers. HE participated in developing sampling protocols, overseeing the field data collection, and provided comments on the manuscript. KI contributed to interpreting results and provided edits and comments to the manuscript. TM oversaw the method development, implementation, and data analysis and contributed to drafting and editing the manuscript. All authors participated in interpretation of the results and all authors read and approved the final manuscript.

## Supplementary Material

Additional file 1: Table S1 Details on GIS-based source indicators. Click here for file
