# Supplementary material for: Spatial variability in levels of benzene, formaldehyde, and total benzene, toluene, ethylbenzene and xylenes in New York City: a land-use regression study
Source: Environ Health. 2012 Jul 31;11:51. doi: 10.1186/1476-069X-11-51 (PMC3420325; doi:10.1186/1476-069X-11-51)
Supplement: Additional file 1: Table S1 — Details on GIS-based source indicators. [file 1476-069X-11-51-S1.pdf]

**Supplemental Table 1 - Details on GIS-based source indicators**

| Source Category | Data Source | Resolution/positional accuracy | Links to Sources |
|---|---|---|---|
| Traffic Indicators | New York Metropolitan Transportation Council (NYMTC) traffic data,2005 | Link level data assigned to street centerline data generated by Tele Atlas | http://www.nymtc.org/ |
| | U.S. Federal Highway Administration Highway Performance Monitoring System (HPMS) data, 2007 | Data linked to National Highway Planning Network line file; accuracy +- 80m | www.bts.gov/publications/national_transportation_atlas_database/2011/ |
| | Accident Location Information System (ALIS) road network data, 2008 | +/- 40 ft | http://gis.ny.gov/gisdata/inventories/details.cfm?DSID=932 |
| | MPSI TrafficMetrix ™ data, 1989-2006 | Linked to ALIS road network | www.mpsisolutions.com |
| | NYC Department of Transportation (DOT) traffic signals, truck and bus routes, 2008 | Truck and bus routes aligned to aerial imagery with +/-2 ft resolution; traffic signals geocoded using NYC Dept of City Planning Geosupport, no statement regarding positional accuracy | http://a841-dotweb01.nyc.gov/datafeeds/ |
| Population Metrics | U.S. Census Bureau 2000 data | Census block | http://factfinder2.census.gov/faces/nav/jsf/pages/index.xhtml |
| | Oak Ridge National Laboratory LandScan™ data, 2006 | 3 arc second | http://www.ornl.gov/sci/landscan/ |
| Built Space | NYC Department of City Planning Primary Land Use Tax Lot Output (PLUTO™) data, 2007 | 1:250 ft | http://www.nyc.gov/html/dcp/html/bytes/applbyte.shtml |

| | | | |
|---|---|---|---|
| Permitted Emissions | NYS Department of Environmental Conservation (DEC) permit data, 2005 | No statement regarding positional accuracy | http://www.dec.ny.gov/chemical/68524.html |
| | NYC Department of Environmental Protection (DEP) permit data, 2008 | Geocoded using NYC Dept of City Planning Geosupport, no statement regarding positional accuracy | http://www.nyc.gov/html/dep/html/air/index.shtml |
| | NYC Department of Buildings (DOB) permit data, 2008 | Geocoded using NYC Dept of City Planning Geosupport, no statement regarding positional accuracy | http://www.nyc.gov/html/dob/html/bis/bis.shtml |
| Transportation Facilities | NYC Department of Citywide Administrative Services, 2008 | Geocoded using NYC Dept of City Planning Geosupport, no statement regarding positional accuracy | https://nycopendata.socrata.com/Facilities-and-Structures/IPIS-Integrated-Property-Information-System-/n5mv-nfpy |
| | NYC Department of Education, 2008 | Geocoded using NYC Dept of City Planning Geosupport, no statement regarding positional accuracy | http://schools.nyc.gov/default.htm |
| Distributed Facilities | NYC Department of Sanitation inspections of private waste transfer stations | Geocoded using NYC Dept of City Planning Geosupport, no statement regarding positional accuracy | http://www.nyc.gov/html/dsny/html/home/home.shtml |
| | NYC Department of Citywide Administrative Services,2008 | Geocoded using NYC Dept of City Planning Geosupport, no statement regarding positional accuracy | https://nycopendata.socrata.com/Facilities-and-Structures/IPIS-Integrated-Property-Information-System-/n5mv-nfpy |
| Land cover | USGS impervious surface, 2001 | 85 ft resolution | http://seamless.usgs.gov/imperv.php |
| | NYC Department of Parks EMERGE dataset, 2001 | 3 ft resolution | http://www.nycgovparks.org/ |
| Built/natural topography | NYC DOITT building heights,2009 | +/- 2 ft | http://www.nyc.gov/html/doitt/html/citywide/gis_downloads.shtml |
| | NYC DOITT elevation DEM, 2006 | 1 ft | http://www.nyc.gov/html/doitt/html/citywide/gis_downloads.shtml |
